# A Simple Zn^2+^ Complex-Based Composite System for Efficient Gene Delivery

**DOI:** 10.1371/journal.pone.0158766

**Published:** 2016-07-19

**Authors:** Zhe Zhang, Yanjie Zhao, Xianggao Meng, Dan Zhao, Dan Zhang, Li Wang, Changlin Liu

**Affiliations:** Key Laboratory of Pesticide and Chemical Biology, Ministry of Education and School of Chemistry, Central China Normal University, Wuhan, 430079, China; Martin-Luther-Universität Halle-Wittenberg, GERMANY

## Abstract

Metal complexes might become a new type of promising gene delivery systems because of their low cytotoxicity, structural diversity, controllable aqua- and lipo-solubility, and appropriate density and distribution of positive charges. In this study, Zn^2+^ complexes (**1–10**) formed with a series of ligands contained benzimidazole(bzim)were prepared and characterized. They were observed to have different affinities for DNA, dependent on their numbers of positive charges, bzim groups, and coordination structures around Zn^2+^. The binding induced DNA to condensate into spherical nanoparticles with ~ 50 nm in diameter. The cell transfection efficiency of the DNA nanoparticles was poor, although they were low toxic. The sequential addition of the cell-penetrating peptide (CPP) TAT(48–60) and polyethylene glycol (PEG) resulted in the large DNA condensates (~ 100 nm in diameter) and the increased cellular uptake. The clathrin-mediated endocytosis was found to be a key cellular uptake pathway of the nanoparticles formed with or without TAT(48–60) or/and PEG. The DNA nanoparticles with TAT(48–60) and PEG was found to have the cell transfection efficiency up to 20% of the commercial carrier Lipofect. These results indicated that a simple Zn^2+^-bzim complex-based composite system can be developed for efficient and low toxic gene delivery through the combination with PEG and CPPs such as TAT.

## Introduction

Although nucleic acid delivery mediated by the nonviral carriers including cationic lipids and organic polymers provides a major contribution to development of gene therapy [1–3], the inorganic systems designed for efficient nucleic acid delivery have attracted great interest [4–7].Of inorganic carriers, metal complexes might become one of the promising nonviral gene carriers, because of their low cytotoxicicty, structural diversity, controllable aqua- and lipo-solubility, and appropriate density and distribution of positive charges.

The metal complexes are a promoting agent in efficient nucleic acid condensation. Indeed, in 1980, the complex [Co(NH_3_)_6_]^3+^ had been found to convert relaxed DNAs into nanoparticles with different sizes and morphologies under nearly physiological conditions [8–20]. Recently, the binding of antitumor polynuclear Pt(II) complexes to DNA was observed to lead to DNA condensation likely in a sequence-specific manner via the competition with naturally occurring DNA condensing agents including polyamines under neutral conditions [21]. The mono- and multi-nuclear Ni(II) and Ru(II) complexes with polypyridines were also reported to be an effectively promoting agent in DNA condensation under neutral and acidic conditions [22–26]. The spherically nanosized coordination compoundPd_12_L_24_, which possesses 24 positive charges and mimics a histone octamer in size and charge density, triggers a stepwise condensation process of DNA in a manner similar to that of the natural system [27]. Obviously, these metal complexes promote DNA packing mainly via neutralizing the negative charges on DNA surfaces [28].

A lot of metal complexes that efficiently promote DNA packing were tested at cellular and mouse levels. The DNA nanoparticles formed with two kinds of Ru(II)-polypyridine complexes could be found in cytosol, and the assays by measurements of luciferase activity and fluorescence of green fluorescent protein (GFP) indicated successful expression of the genes released from the nanoparticles. These Ru(II) complexes were also observed to be low cytotoxic [24–26]. Moreover, the genes transferred into cells by the reducible polymers that were linked to Cu(II) complexes also exhibited efficient expression [29]. The transfection activity of the DNA condensates formed with the ferrocenes modified with cationic lipids was observed to be dependent on the redox states of the ferrocences [30–34]. In addition, nanoscale metal-organic frameworks were found to be capable of protecting small interfering RNAs (siRNAs) from nuclease degradation and promoting siRNAs escapes from endosomes to silence multiple drug resistance genes in cisplatin-resistant ovarian cancer cells [35]. The platforms for efficient siRNAs delivery into both cells and mice have also been assembled, respectively, by the Zn^2+^ complex-functionalized nanoconjugates and by ferrocenyl lipids [36,37].

We have reported the finding that the metal complexes formed with a series of ligands contained benzimidazole (bzim) groups are becoming a new type of the nonviral gene delivery systems prone to structural alteration and chemical tailoring [38,39]. The DNA condensation via interactions with these complexes was observed to be driven by both electrostatic attractions between the DNA molecules and the complexes and intermolecular π-πinteractions of the complexes [40–42]. The characteristics of the complexes including their numbers of positive charges and bzim groups, as well as coordination geometries around the metal ions, were found to have a strong correlation both with their DNA binding and cytotoxicity, and with the formation, features and cell transfection of their DNA condensates [40–46]. Although the addition of a nuclear localization sequences (NLS) can significantly improve the cell transfection efficiency of the DNA condensates, expression of the genes transferred by the complexes is poor when compared with that of the genes by the commercial carrier Lipofect [38,39,43–46]. In addition, the complexes with either Cu^2+^or Co^2+^ are high toxic through the redox activity of the divalent metal ions, and those with Ca^2+^ are unstable in cell cultures [40–46]. Therefore, to overcome the disadvantages of the Cu^2+^, Co^2+^ and Ca^2+^ complexes, a series of bzim complexes with redox-inactive Zn^2+^ were synthesized and characterized here, and a simple Zn^2+^ complex-based composite system for efficient and safe gene delivery was found through the combination of a cell-penetrating peptide TAT(48–60) and polyethylene glycol (PEG).

## Materials and Methods

### Materials

The plasmid pBR322 DNA and calf thymus DNA (ctDNA) were from Takara, the plasmid pGL3 control vector and dual luciferase reporter gene assay kid were from Promega. Unless otherwise stated, the concentrations of DNAs were expressed in base pairs. The cell-penetrating peptide TAT(48–60) (GRKKRRQRRRPQ) was from Shanghai ABBiochem. Chlorpromazine hydrochloride, amiloride hydrochloride, genistein, Lipofectamine^™^ 2000 (Lipofect), 3-(4,5-dimethylthiazol-2-yl)-2,5-diphenyltetrazolium bormide (MTT), polyethylene glycol (PEG, Mw 3350) and 4,6-diamino-2-phenylindole (DAPI) were purchased from Sigma. All other chemicals were purchased from chemical suppliers and directly used without further purification. All samples were prepared using distilled water that had been passed through a Millipore-Q ultrapurification system. African green monkey cell line COS 7 was obtained from China Center of Typical Culture Collection. The cell culture Dulbecco’s modified Eagle’s medium (DMEM) and fetal bovine serum (FBS) were from Gibco.

### Synthesis and characterization

The Zn^2+^-bzim complexes **1–10** were prepared and characterized according to the reported synthesis programs [40–46], details could be found in the Supplementary Information.

### Determination of DNA affinity

The affinities (*K*_d_) of the Zn^2+^-bzim complexes for ctDNA were determined by UV–vis absorption titrations [41]. All reactions containing each Zn^2+^-bzim complex (40 μM) and ctDNA of increased concentrations were incubated for 2 min at 37°C in pH 7.4, 20 mM Tris-HCl buffer, and the absorption spectra (230–300 nm) of the resulted solutions were measured with an analytic jena SPECORD 210 spectrophotometer.

### Characterization of DNA condensation

The reactions containing pGL3 or pBR322 of given amounts and each Zn^2+^-bzim complex of varied concentrations were incubated for 60 min at 37°C, respectively, in the absence and in the presence of TAT(48–60) and/or PEG in pH 7.4, 20 mM Tris-HCl buffer prior to characterization. The resulted DNA condensates were immediately examined with electrophoresis mobility shift assays (EMSA) and transmission electron microscope (TEM) [40,41]. On the one hand, 50 μM pGL3or pBR322 DNA was incubated for 60 min at 37°C with each Zn^2+^-bzim complex of 0–250 μM in the buffer for EMSA experiments. Electrophoresis of the resulted DNA condensates was carried out using 1% agarose gel with 20 μg ethidium bromide in TAE (40 mM Tris-acetate and 1 mM EDTA, pH 8.2) running buffer. On the other hand, aliquots of the ongoing DNA condensation reaction mixtures were taken at different time points of incubation or concentration points of reactants for TEM visualization. Samples were placed 20 μL each time on a freshly glow-discharged carbon-coated grid, absorbed for 2 min, and then washed the grid with deionized water for imaging. Grids were directly imaged on a Tecnai G2 20 TEM operating at 200 kV.

### Cytotoxicity assays

First, 50 μM plasmid DNAs were incubated for 60 min at 37°C with each Zn^2+^-bzim complex at the complex/DNA ratios of 1:1, 2:1, 3:1 and 4:1 in pH 7.4, 20 mM Tris-HCl buffer. Following centrifugation, the DNA nanoparticles were collected from the reactions and suspended in an FBS-free DMEM for their evaluation of cytotoxicity by MTT assays [40,45]. Then, COS 7 cells were seeded at about 4000–40 000 cells each well in 96-well plates, and maintained at 37°C in a 5% CO_2_ humidified air atmosphere until growth reached 80% confluence as a monolayer. The cells were incubated in the atmosphere for 4 h following replacement of the FBS-containing medium with an FBS-free one. 50 μL solutions of each complex and suspensions of the freshly prepared DNA nanoparticles were added, respectively, into wells. After incubation of 24 h, 10 mL of MTT (5 mg/mL) was added to the wells. After re-incubation for 4 h, the MTT-containing medium was replaced by 150 mL DMSO. Finally, the 96-well plates were oscillated for 15 min to fully dissolve the formazan crystal formed by living cells. The relative viability of the cells in each well was obtained by determining the absorbance at 490 nm of each well with the Biotek Synergy^™^ 2 Multidetection Microplate Reader. Untreated cell (in DMEM) were used as control, and the relative viability of cells exposed, respectively, to the complexes and condensates (mean% ± SD, n = 3) was expressed as OD_sample_/OD_control_× 100%. The analysis of data was carried out using software ORIGIN.

### Cell transfection

pGL3 control vector contains SV40 promoter and enhancer sequences, and strongly expresses firefly luciferase, and was used in preparation of DNA condensates to examine cell transfection of the condensates. Luciferase’s substrate (luciferin) exhibits yellow luminescence (the largest emission at 590 nm) upon oxidation. First, to prepare condensates of the vector, 30 μM pGL3 plasmid was incubated for 60 min at 37°C with each Zn^2+^-bzim complex at the complex/DNA ratios of 1:1, 2:1, 3:1 and 4:1, respectively, in the absence and in the presence of TAT(48–60) (TAT/DNA = 0.8:1) and/or PEG (PEG/DNA = 33:1) in pH 7.4, 20 mM Tris-HCl buffer. The transfection system utilizing the commercial gene delivery agent Lipofect was designed for comparison under the conditions tested (DNA/TAT/PEG = 1:0.8:33). Lipofect-plasmid complexes were prepared at the optimal weight ratio of 4:1 for cell transfection according to the manufacture’s instructions. The condensates were collected from the reactions following centrifugation and suspended in an FBS-free DMEM for their evaluation of cell transfection [40,45]. COS 7 cells were seeded at about 4000–40 000 cells each well in 96-well plates, and maintained at 37°C in a 5% CO_2_ humidified air atmosphere until growth reached 80% confluence as a monolayer. The cells were incubated in the atmosphere for 4 h following replacement of the FBS-containing medium with an FBS-free one. 200 μL suspensions of the freshly prepared DNA condensates were added into wells, and the cells were incubated for 24 h in the atmosphere. The cells exposed to the pGL3 condensates were treated for 30 min at 4°C in end-over-end rotation with the reporter gene lysis buffer (50 mM Tris-HCl, pH 7.5, 150 mM NaCl, 2% Triton X-100, 2% NP40). After lysis, cell debris was separated by centrifugation for 5 min at 15,000 rpm and 4°C, and supernatants were collected for luciferase activity assays. The luciferase activity was expressed as relative luminescence units (RLU)/mg proteins recorded at 590 nm for lysates of the cells exposed to the DNA nanoparticles using a Biotek Synergy^™^ 2 Multi-detection microplate reader according to the manufacturer’s instructions of luciferase kit. To remove the background and artifacts, the blank controls were set in these experiments. The amount of proteins in each transfection lysate was measured using a Bio-Rad RC-DC protein assay kit according to the manufacturer’s instructions.

### Cellular uptake pathways

The DNA condensates (pGL3-8, pGL3-TAT(48–60)-8 and pGL3-TAT (48–60)-8-PEG) for determination of cellular uptake pathways were prepared at the DNA/TAT/**8**/PEG ratio of 1:0.8:1:33, as in transfection experiments. Meanwhile, the condensates were also prepared using DAPI-stained ctDNA for observations with fluorescence microscope. The endocytosis inhibitors used to treat cells were fixed at 3 μg/mL because the viability of cells maintained > 90% at this dose. Therefore, 3 μg/mL of chlorpromazine hydrochloride, amiloride hydrochloride and genistein was added, respectively, into the wells containing the COS 7 cells cultured in the FBS-free DMEM. Following incubation for 24 h, 200 μL suspensions of the freshly prepared DNA condensates were added into the wells, and the cells were incubated for 24 h in the atmosphere. Then, on the one hand, the cells exposed to the DAPI-stained ctDNA condensates were observed with a Leica DMI 3000B inverted fluorescence microscope. On the other hand, the luciferase activity was measured, respectively, for the inhibitor-treated and -untreated cells according to the programs described in Cell Transfection Experiments.

## Results and Discussion

### Structures of the Zn^2+^-bzim complexes and their DNA binding

**Fig 1:**
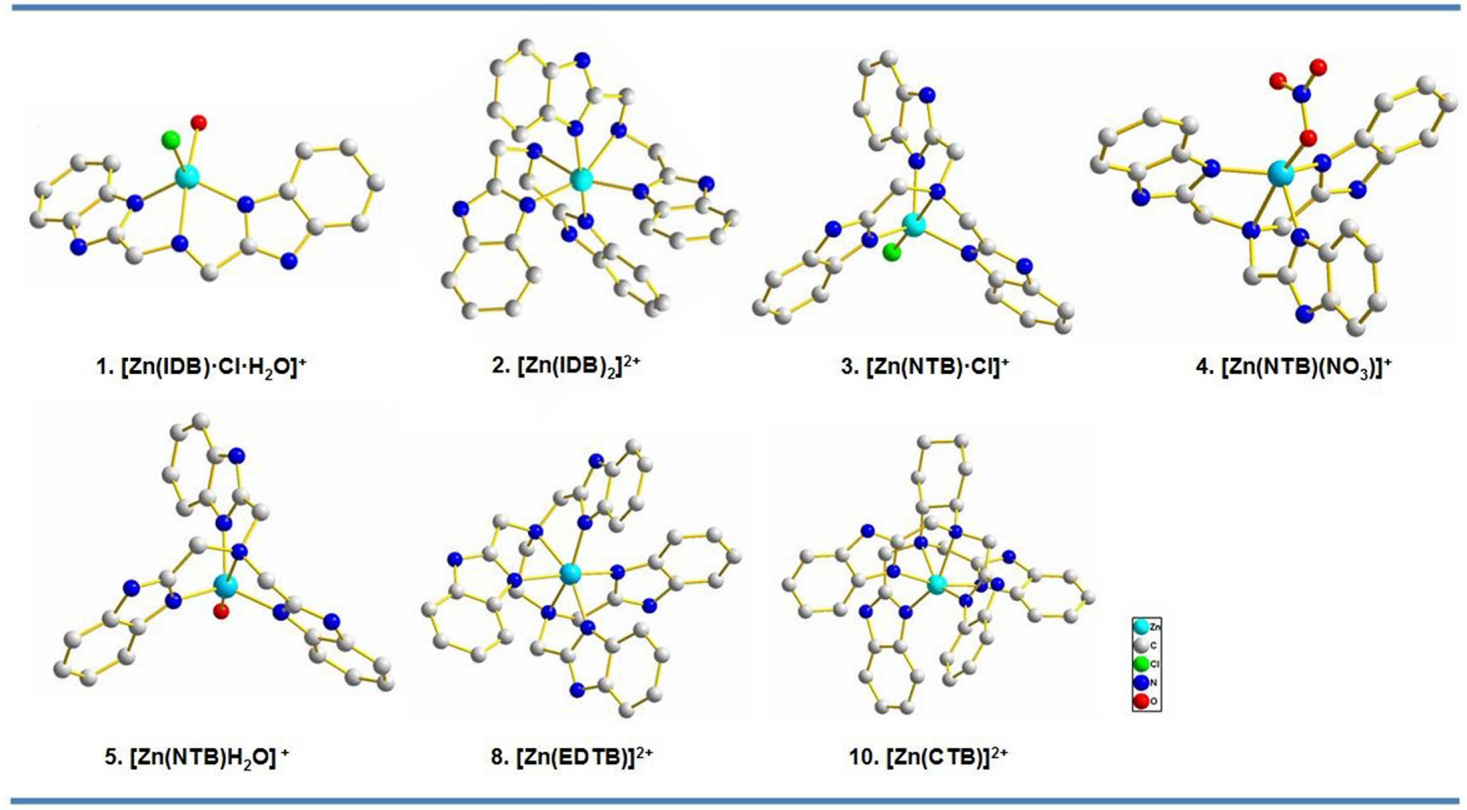
Structures of cations of the complexes 1–5, 8 and 9. Solvent molecules and counteranions were omitted for clarity, and all atoms were shown as sphere of arbitrary diameter.

The Zn^2+^-bzim complexes **1–10** were synthesized and characterized according to a program similar to the synthesis of other metal-bzim complexes (S1 Text) [40–46]. The complexes have the octahedral or trigonal bipramidal coordination around the Zn^2+^ ion and one or two positive charges, dependent on the numbers of bzim groups in the ligands and counteranions (Table 1 and Fig 1).

**Table 1:** Structural features and affinity (*K*_d_) for ctDNA of the Zn^2+^-bzim complexes.

		Coordimationpolyhrdron around Zn^2+^	Number of positive charges	Number of bzim groups	*K*_d_, μM
1	**[Zn(IDB)Cl(H**_**2**_**O)]Cl**	**trigonal bipyramid**	**1+**	**2**	**3.45**
2	**[Zn(IDB)**_**2**_**](ClO**_**4**_**)**_**2**_	**octahedron**	**2+**	**4**	**5.26**
3	**[Zn(NTB)Cl]Cl**	**trigonal bipyramid**	**1+**	**3**	**2.17**
4	**[Zn(NTB)(NO**_**3**_**)]NO**_**3**_	**octahedron**	**1+**	**3**	**1.32**
5	**[Zn(NTB)H**_**2**_**O](ClO)**_**4**_	**trigonal bipyramid**	**2+**	**3**	**1.11**
6	**[Zn(EDTB)]Cl**_**2**_	**octahedron**	**2+**	**4**	**1.54**
7	**[Zn(EDTB)](NO**_**3**_**)**_**2**_	**octahedron**	**2+**	**4**	**1.42**
8	**[Zn(EDTB)](ClO**_**4**_**)**_**2**_	**octahedron**	**2+**	**4**	**0.32**
9	**[Zn(CTB)](NO**_**3**_**)**_**2**_	**octahedron**	**2+**	**4**	**2.13**
10	**[Zn(CTB)](ClO**_**4**_**)**_**2**_	**octahedron**	**2+**	**4**	**1.64**

The binding equilibrium constants between each of the Zn^2+^-bzim complexes and calf thymus DNA (ctDNA) were determined by ultraviolet and visible (UV-vis) absorption titrations (S1 Fig). The data showed that the affinities of the complexes for the DNA are at a micro-molar scale with an exception of **8** whose DNA affinity is higher than those of the other Zn^2+^ complexes (Table 1), indicating that the DNA binding of the complexes is dependent on their numbers of positive charges and bzim groups, as well as coordination geometries of Zn^2+^ [40–46]. As observed for the bzim complexes with Co^2+^ [46], the asymmetric structure of **8** facilitates its binding to DNA compared to the symmetric structure of **2**. In addition, the counteranions in the complexes 6–8 were observed to have an impact on the DNA binding. The main driving forces in the DNA binding are also electrostatic interactions between the complexes and the DNA, and π–π interactions between DNA bases and bzim groups owing to the intercalation of the bzim groups into DNA base pairs [40–42].

### Characterization of DNA condensation

The roles of the Zn^2+^-bzim complexes in DNA condensation were examined at pH 7.4 through both EMSA in agarose gels and TEM. The EMSA results showed that the ability of the complexes to induce condensation of the supercoiled DNA was enhanced in the order of **1**~**2**~**6**<7~**9**~**10**<**3**~**4**~**5**~**8** at the tested molar ratios of complex/DNA, and the DNA condensation became more pronounced with increasing the ratios of complex/DNA (S2 Fig). The complexes **1** and **2** were not found to be capable of efficiently inducing the DNA condensation in the range of complex/DNA ratios tested. These qualitative results indicated that the DNA-condensing ability can be correlated not only with the π–π interactions between bzim groups in the complexes, but also with the affinities of the complexes for DNA (Table 1), as observed for the Co^2+^-bzim complexes [40–46].

**Fig 2:**
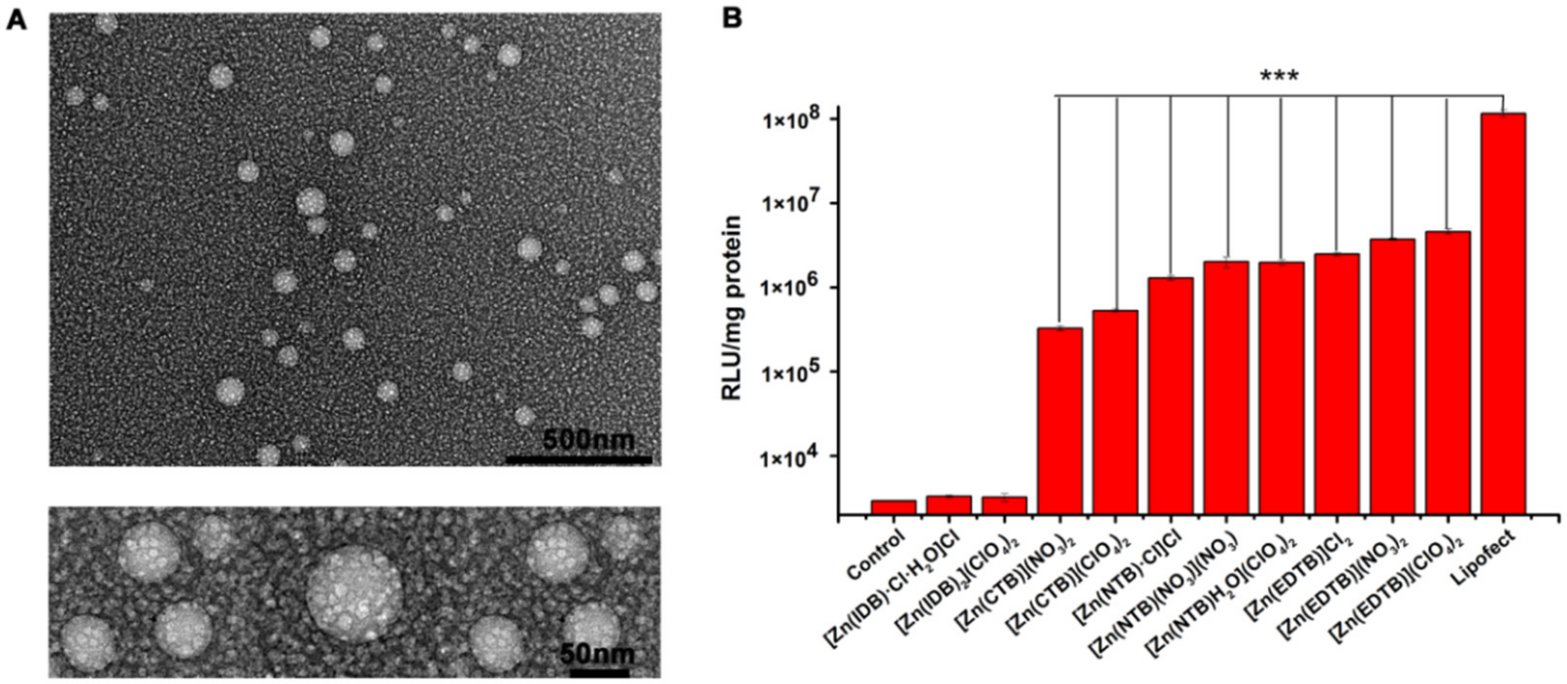
(A) TEM images of the DNA condensates provided by 8. 5 μM pGL3 DNA was incubated for 60 min with 10 μM 8 at 37°C in pH 7.4, 20 mM Tris-HCl buffer prior to TEM imaging. (B) Cell transfection of the DNA condensates. The cell transfection efficacy was expressed by the luciferase activity measured in RLU/mg protein. The condensates were prepared at 2:1 of Zn^2+^-bzim complex/DNA under the conditions tested. The control was the untreated DNA. The Lipofect-plasmid complex was prepared at the optimal weight ratio of 4:1 for comparison. n ≥ 3, ***P = 0.001.

The above-mentioned EMSA results indicated that the DNA condensation was observed to be significant for the complexes **3**–**10** when the ratios of complex/DNA were ≥2 (S2 Fig). Thus, the DNA condensates formed with **8** at **8**/DNA of 3:1 were selected for TEM observations because this complex has both the strongest affinity for DNA. The TEM images showed that the monodispersed DNA condensates were individual and compact spherical nanoparticles with a diameter of ~ 50 nm (Fig 2A), revealing that the Zn^2+^-bzim complex is an efficient DNA-packing agent.

### Cell transfection

Prompted by the morphological observations that indicated that the DNA nanoparticles formed with the Zn^2+^-bzim complexes have a nano-scale size suitable for entry into cells, the cell transfection efficiency of the DNA nanoparticles was evaluated by measuring the luciferase activity in COS 7 cells. The luciferase activity in the COS 7 cells exposed to the untreated DNA was also measured for comparison under the conditions tested.

Cytotoxicity of the DNA nanoparticles was determined by MTT assays prior to the evaluation of cell transfection. First, the cells exposed to each Zn^2+^-bzim complex of different concentrations (0–100μM) for 24 h maintained more than 80% of viability, and the IC_50_ values (the concentrations required to kill 50% of the cells) of the complexes can not be exactly obtained, but were estimated to be > 100μM, indicating that the complexes are low toxic. Then, the nanoparticles were prepared at the complex/DNA ratios of 1:1, 2:1, 3:1 and 4:1 for the MTT estimation of cell viability under the conditions tested. The data showed that the viability of the cells exposed to these nanoparticles was also higher than 80% up to the complex/DNA ratio of 4:1, although the cell viability was slightly reduced with increasing complex/DNA ratios (S3 Fig), indicating the slight cytotoxicity of the DNA nanoparticles has not observable impact on cell growth. The low toxicity of both Zn^2+^-bzim complexes and their nanoparticles with DNA could be ascribed to the redox-inactivity of Zn^2+^.

The luciferase activity (RLU/mg proteins) was measured for lysates of the cells exposed to the DNA nanoparticles (Fig 2B). First, the data of luciferase activity were used to examine the impact of complex/DNA ratios on the expression of the luciferase gene transferred into the cells. The results showed that the complexes **3**–**8** had much higher RLU values than that of the control at the DNA/complex ratios tested and reached the largest RLU values, respectively, at the complex/DNA ratios of 2:1 and 3:1. However, the values of **1** and **2** were not significantly higher than that of the control, and the values of **9** and **10** were also two orders of magnitude higher than that of the control, but at least one order of magnitude lower than those of **3**–**8** (S4 Fig). This result indicated that the complexes **3**–**8** can release genes of interest into the cells. Then, the luciferase activity was further determined for the cells exposed, respectively, to each complex at the complex/DNA ratio of 2:1 and to the commercial gene carrier Lipofect at the optimal weight ratio of 4:1 (Fig 2B). The data showed that (1) **1** and **2** did not significantly elevate the luciferase activity in the cells relative to the control, (2) the RLU values of **3**–**8** reached 2–5% of that of Lipofect and increased in the order of **3**<**4**<**5**<**6**<**7**<**8**, and (3) **8** possessed the highest transfection efficacy at the complex/DNA ratio of 2:1. These results indicated that the complexes **3**–**8** are an efficient gene-delivering agent at the tested complex/DNA ratios.

The above-measured cell transfection efficacy can be understood based on both the DNA affinity of the Zn^2+^-bzim complexes and their structural characteristics (Table 1). According to their *K*_d_ values, the DNA affinity of the complexes can be divided into four groups in the order of binding strength: **1**, **2**<**3**, **9**<**4**–**7**, **10**<**8**, whereas the complexes can also be categorized into four groups in the order of luciferase activity: **1**, **2** <**9**, **10**<**3**–**7**<**8** (Fig 2B), i.e., the increasing order in the cell transfection of the DNA condensates is consistent with the increased DNA affinity order of the complexes. This indicated that the binding interactions between the complexes and DNA play a key role in cell transfection of the DNA nanoparticles, as observed for the bzim complexes with the other metal ions [46].However, it is noteworthy that the interactions of the complexes with DNA are dependent on their numbers of positive charges and bzim groups, as well as the coordination structures around Zn^2+^.

### A Zn^2+^-bzim complex-based composite system

Although the Zn^2+^-bzim complexes **3**–**8** can release genes of interest into cells, the transfection efficiency of their condensates is much lower than that of Lipofect (Fig 2B). The presence of the peptides with multiple positive charges has been reported to be capable of significantly improving the gene-delivering function of the nonviral carriers. In fact, the addition of the peptide NLS with four positive charges can not only decrease the amount of the Co^2+^ complexes requisite for the efficient DNA condensation, but also elevate the transfection efficiency of their DNA condensates [45]. Hence, to upregulate the expression of the gene transferred by the Zn^2+^-bzim complexes, a composite system was prepared by combining the complex **8**, the cell-penetrating peptide TAT(48–60) and PEG, because the condensates with **8** were selected because of its highest transfection efficiency among the complexes tested. TAT(48–60) with **8** positive charges is a polypeptide from human immunodeficiency virus type 1 TAT [47]. TAT(48–60) and PEG have extensively been used to significantly improve the gene-delivering ability of different kinds of nonviral carriers [47,48].

**Fig 3:**
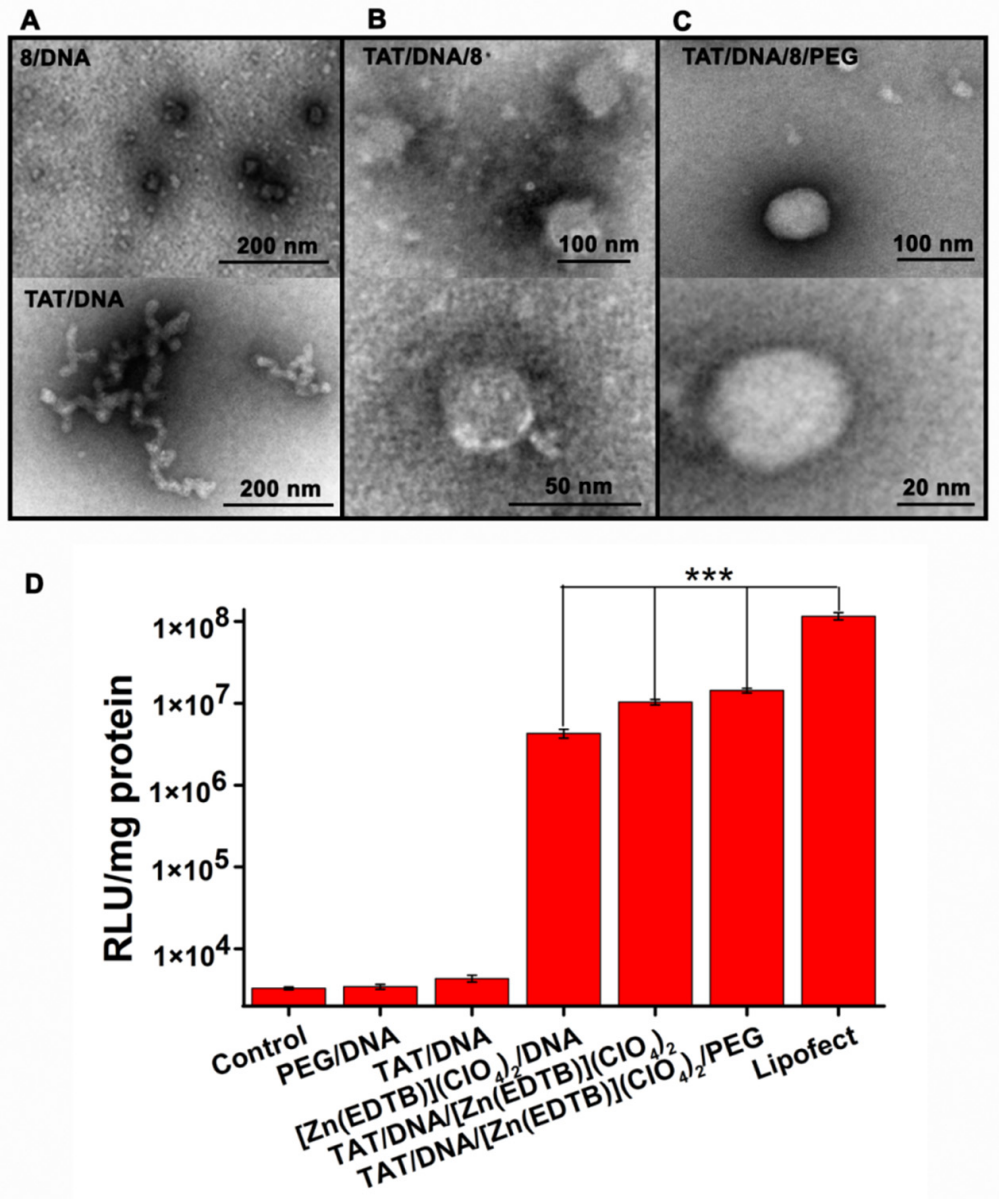
TEM images (A–C) and cell transfection experiments of DNA condensates (D). The DNA condensates were prepared, respectively, by incubating 3 μM pGL3 DNA with 2.4 μM TAT(48–60) (A), 3 μM pGL3 DNA with 2.4 μM TAT(48–60) plus 3 μM **8** (B), 3 μM pGL3 DNA with 2.4 μM TAT(48–60) plus 3 μM **8** plus 33 μM PEG (calculated according to its average molecular weight) (C) for 60 min at 37°C in pH 7.4, 20 mM Tris-HCl buffer. (D) Comparison of the cell transfection efficiency of the systems consisted of different components. For the preparation of condensates, 30 μM pGL3 plasmid was incubated for 60 min at 37°C with **8** at **8**/DNA ratios of 1:1, 2:1, 3:1 and 4:1 in pH 7.4, 20 mM Tris-HCl buffer. The 4:1 Lipofect-pGL3 DNA complex was prepared at the weight ratio of DNA/Lipofect = 4:1 and at the molar ratio of DNA/TAT/PEG = 1:0.8:33 under the conditions tested. n ≥ 3, ***P = 0.001.

The morphology of DNA condensates was first examined in the presence of TAT(48–60) and PEG. Our previous work showed that the amount of the metal complexes required for the efficient DNA condensation can be significantly decreased because of the addition of the peptide with multiple positive charges [45]. In fact, the DNA condensation monitored by light scattering indicated that the optimal amounts of **8**, TAT(48–60) and PEG requisite for the efficient DNA packing were at the DNA/TAT/**8**/PEG ratio of 1:0.8:1:33. The TEM images showed that the DNA in the complex with TAT(48–60) at the DNA/TAT ratio of 1:0.8 remained a relaxed state, indicating that this peptide can neutralize the negative charges on DNA surfaces, but cannot promote the conversion of DNA into a nanoparticle (Fig 3A). The sequential addition of TAT(48–60) and **8** led to the conversion of DNA into nanoparticles with a diameter of ~ 50 nm under the conditions tested (Fig 3B), indicating that **8** and TAT(48–60)could cooperatively promote DNA condensation, but the DNA condensation is mainly dependent on the addition of **8**, because **8** was added to the DNA condensation reaction following this peptide, and the DNA nanoparticles formed with and without TAT(48–60) had the almost same diameters (Figs 2A and 3B).The introduction of PEG converted these DNA nanoparticles into spherical and compact condensates whose diameters were ~ 100 nm, and the large nanoparticles did not conglomerate and change their profiles and sizes with prolonging incubation time in the buffer used (Fig 3C). This increase in diameters of the DNA condensates indicated that PEG binds only at surfaces of the nanoparticles. These results revealed that (1) the complex provides a pivotal contribution to the condensation of DNA compared with the CPP and PEG, (2) the co-presence of **8** and TAT(48–60) results in the formation of loose and irregular DNA condensates compared with those formed only with **8** (Fig 2A) likely because of the electrostatic repulsion among these DNA-bound TAT(48–60) molecules with multiple positive charges, and (3) the DNA condensates formed in the composite system containing PEG are more compact, larger and more stable than those formed in the DNA condensationsystems only containing either **8** or **8** and TAT(48–60) in the buffer tested. Obviously, these features of the DNA condensates formed with multi-components should facilitate their cell transfection.

Then, the transfection efficiency was compared by measuring the luciferase activity in the cells treated with the DNA condensates consisted of different components. The condensates for transfection assays were prepared at the DNA/TAT/**8**/PEG ratio of 1:0.8:1:33, as in TEM imaging. For comparison, the lipid–DNA nanoparticles were prepared at the DNA/Lipofect weight ratio of 4:1 and at the DNA/TAT/PEG molar ratio of 1:0.8:33 under the conditions tested. The dada showed that the luciferase activity in the cells treated, respectively, with the DNA-TAT(48–60) or DNA-PEG complex approached that in the control, indicating that the systems without **8** cannot transfer the gene into cells (Fig 3D). The transfection efficiency of the DNA condensates formed with **8** reached 5% of Lipofect according to their data of RLU/mg proteins, as observed in transfection experiments of the Zn^2+^-bzim complexes alone (Fig 2B). The combination of TAT(48–60) and **8** resulted in a 2-fold increase in transfection efficiency relative to the condensate formed with **8** (Fig 3D), indicating that the addition of TAT(48–60)might facilitate entry of the DNA nanoparticles into cells because of its cell-penetrating function (47). The incorporation of PEG further elevated the transfection efficiency to ~ 20% of Lipofect, as indicated by their luciferase activity (Fig 3D), demonstrating that the transfection efficiency of the DNA nanoparticles coated with PEG is comparable to that of the nanoparticles with Lipofect under the conditions tested. This high transfection efficiency can be ascribed to the high stability of and appropriate sizes and profiles of the DNA nanoparticles formed in the composite delivery system (Fig 3C). Thus, the complex **8**-based composite system combined with TAT(48–60) and PEG is a simple system for efficient gene delivery because of its efficient entry into cells and high stability in the culture.

### Cellular uptake pathway

Cellular uptake pathways remained to be explored for the metal-bzim complex-based DNA nanoparticles formed with and without positively charged peptides and PEG, because the efficient cellular uptake is responsible for their high transfection efficiency. The main pathways of nanoparticle entry into cells include dynamin-dependent endocytosis, which are divided into clathrin-mediated endocytosis (CME, for ~ 120 nm) and caveolin-mediated endocytosis (CvME, for ~ 60 nm), and dynamin-independent macropinocytosis (> 1000 nm) [49]. The specific inhibitor are chlorpromazine for CME, genistein for CvME, and amiloride, respectively [49]. Therefore, the key endocytosis pathways of the DNA condensates formed with the Zn^2+^-bzim complexes can be determined by measuring the reduction in the luciferase activity following transfection in the cells treated for 1 h, respectively, with these specific inhibitors prior to transfection. The cellular uptake of the DNA condensates formed in the presence of TAT and PEG might occur mainly via a CME pathway because of their sizes of ~ 100 nm [49].

**Fig 4:**
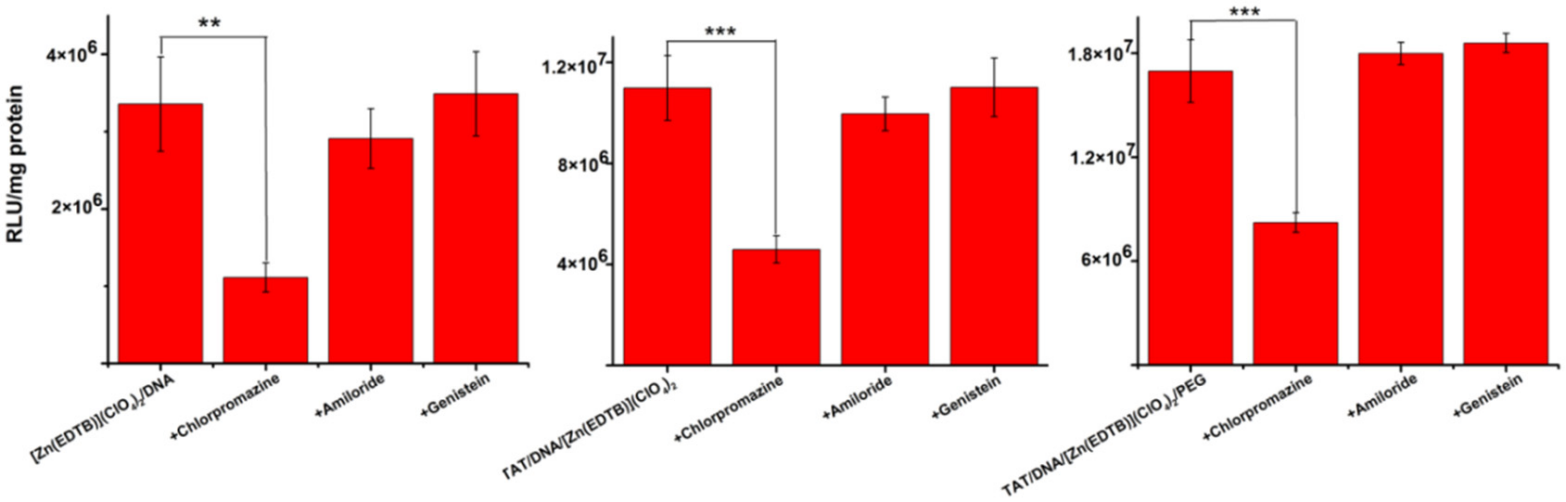
Determination of cellular uptake pathways of DNA condensates. Here, the DNA condensates were prepared as in Cell Transfection Experiments. Cellular uptake pathways of the DNA condensates were determined by the reduction in the luciferase activity of the cells treated by the specific inhibitors. n ≥ 3, **P = 0.01, ***P = 0.00.

In order to support the above-mentioned hypothesis, the luciferase activity was measured in the cells treated with three kinds of endocytosis inhibitors. The data showed that the luciferase activity was not almost reduced in the cells treated with genistein for transfection of the DNA-**8**, DNA-TAT(48–60)-**8** and DNA-TAT(48–60)-**8**-PEG condensates (Fig 4), ruling out the possibility that these condensates enter into cells via CvME. However, the luciferase activity in the cells treated with chlorpromazine was reduced, respectively, to ~ 30%for the **8**-induced condensates (Fig 4, left), to ~ 40% for the condensates formed with TAT(48–60) plus 8 (Fig 4, middle), and to ~ 50% for ones with TAT(48–60) plus **8** and PEG (Fig 4, right) of the cells treated without this inhibitor, indicating that the transfection efficiency of these condensates was significantly reduced when CME was inhibited by chlorpromazine. This result indicated that the cellular uptake of the **8**-based DNA condensates occurs mainly via CME irrespective of the addition of TAT(48–60) and PEG, as expected. In addition, the luciferase activity was also observed to be slightly reduced in the cells treated with amiloride for the condensates that were not coated with PEG (Fig 4, left and middle), suggesting that the inhibition of macropinocytosis can block uptake of the condensates to an observable degree, because macropinocytosis is a key entry pathway of the particles with > 1000 nm into cells[50]. This result implicates that the DNA condensates of ~ 50 nm might conglomerate into large particles over the incubation periods in the culture because of lacking of the PEG coating.

To support the above-performed observations, three kinds of condensates were prepared using the ctDNA stained with the fluorescent dye 4,6-diamino-2-phenylindole (DAPI). These three kinds of DNA condensates emitted blue fluorescence under an inverted fluorescent microscope. The results showed that the cells treated with genistein or amiloride emitted blue fluorescence in all the intracellular regions, and the nucleus profiles were unclear like the control treated without the inhibitors (S5 Fig), indicating the inhibition of both CvME and macropinocytosis can not impact the cellular uptake of these three kinds of condensates. The blue fluorescence was observed only in the regions around nuclei for the cells treated with chlorpromazine (S5 Fig), indicating that the inhibiting CME can block cellular uptake of the condensates. Taken together with the above-mentioned results from the luciferase activity assays, it is concluded that the **8**-based DNA condensates formed, respectively, in the presence and absence of TAT(48–60) or/and PEG are internalized into cells mainly through CME.

## Conclusions

The Zn^2+^-bzim complexes prepared here have different coordination geometries around Zn^2+^. Their affinity for DNA indicated that the asymmetric coordination structure facilitates their binding to DNA in addition to their numbers of their positive charges and bzim groups. The interactions of theses complexes with DNA convert the relaxed DNA into spherical nanoparticles of ~ 50 nm. The cell transfection efficiency of the DNA nanoparticles was poor, although they were low cytotoxic. The addition of the cell-penetrating peptide TAT(48–60) can significantly elevate the cell uptake of the nanoparticles, and the incorporation of PEG can improve the sizes, profiles and stability of the nanoparticles. The clathrin-mediated endocytosis is a key entry pathway into cells of the DNA nanoparticles formed with the Zn^2+^-bzim complex, respectively, in the presence and absence of TAT(48–60) or/and PEG. The gene delivery efficiency by the composite system Zn^2+^ complex-TAT(48–60)-PEG reaches 20% of that by the commercial gene carrier Lipofect. This study indicated that a simple composite system for efficient gene delivery can be obtained by utilization of simple metal complexes with low cytotoxicity. The formation and stability of Zn^2+^-bzim complexes depend on the deprotonation state of the bzim groups, whereas both protonation and deprotonation of bzim groups is sensitive to pH. Because the Zn^2+^-bzim complexes promote DNA condensation at neutral pH, the escape of the DNA condensates from the endosome and the delivery of the gene from the condensates are responsive to pH differences in intracellular organelles. Therefore, this work facilitates development of the new nonviral gene carriers based on simple inorganic complexes.

## Supporting Information

S1 Fig Ultraviolet and visible absorption titrations of each Zn^2+^-bzim complex of 40 μM with ctDNA of increased concentrations. (DOCX)

S2 Fig DNA condensation observed by EMSA in agarose gels. 50 μM pBR322 DNA was incubated for 60 min at 37°C with each Zn^2+^-bzim complex of 0–250 μM in pH 7.4, 20 mMTris-HCl buffer prior to EMSA. Here, the mobility shift was showed to be altered with the ratios of complex/DNA.
(DOCX)

S3 Fig Cytotoxicity evaluation of the Zn^2+^-bzim complex-induced DNA condensates by cell viability. The condensates were prepared, respectively, at the complex/DNA ratios of 1:1, 2:1, 3:1 and 4:1 under the conditions tested. The viability of the COS 7 cells exposed to the condensates was evaluated by MTT assays.
(DOCX)

S4 Fig Dependence of cell transfection of the DNA condensates on the ratios of complex/DNA. The cell transfection efficacy was expressed by luciferase activity measured in RLU/mg protein. The condensates were prepared at 1:1, 2:1, 3:1 and 4:1 of Zn^2+^-bzim complex/DNA under the conditions tested. The control was the untreated DNA. n≥ 3, *P = 0.05, **P = 0.01, ***P = 0.001.
(DOCX)

S5 Fig Observation of cellular uptake pathways of DNA condensates by inverted fluorescence microscope. Here, ctDNA was first stained with the fluorescent dye DAPI. Then, the condensates were prepared using the DAPI-stained ctDNA as in Cell Transfection Experiments. These condensates emitted blue fluorescence under fluorescent microscope.
(DOCX)

S1 Text Synthesis and characterization. (DOCX)
