# Supplementary figures and images for: A Simple Zn2+ Complex-Based Composite System for Efficient Gene Delivery
Source: PLoS One. 2016 Jul 19;11(7):e0158766. doi: 10.1371/journal.pone.0158766 (PMC4951035; doi:10.1371/journal.pone.0158766)

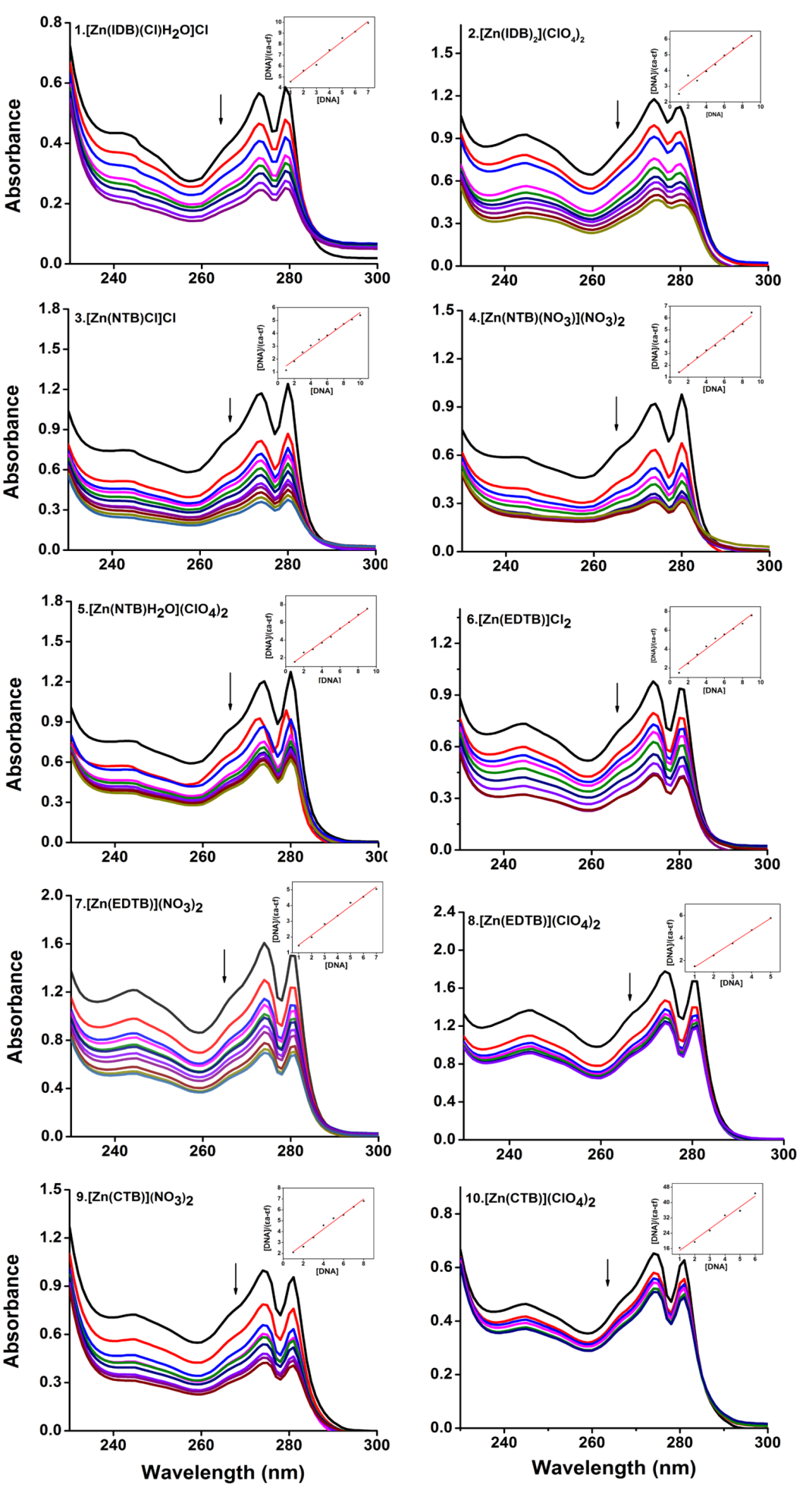

Supplement: S1 Fig — (DOCX) [file pone.0158766.s001.docx]

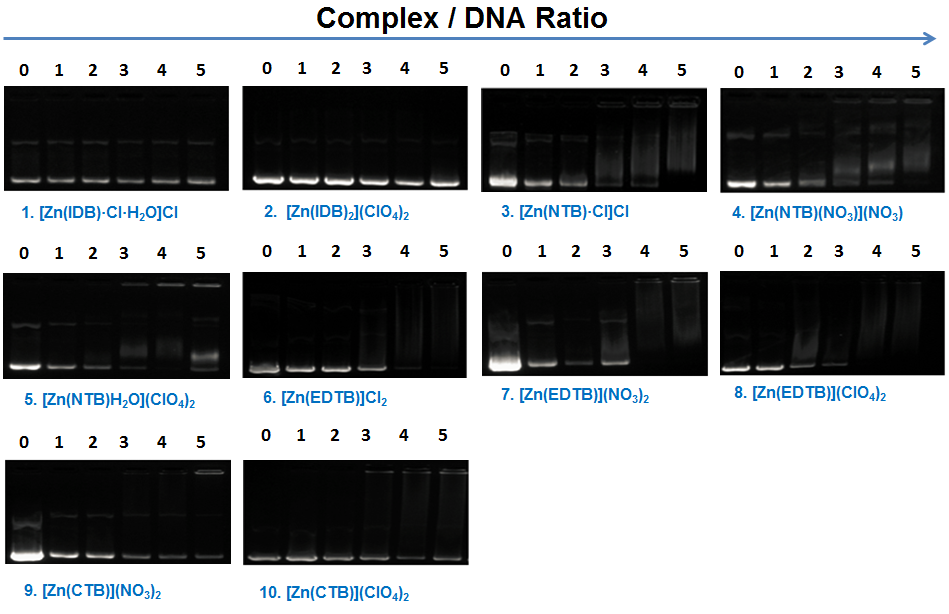

Supplement: S2 Fig — 50 μM pBR322 DNA was incubated for 60 min at 37°C with each Zn2+-bzim complex of 0–250 μM in pH 7.4, 20 mMTris-HCl buffer prior to EMSA. Here, the mobility shift was showed to be altered with the ratios of complex/DNA. (DOCX) [file pone.0158766.s002.docx]

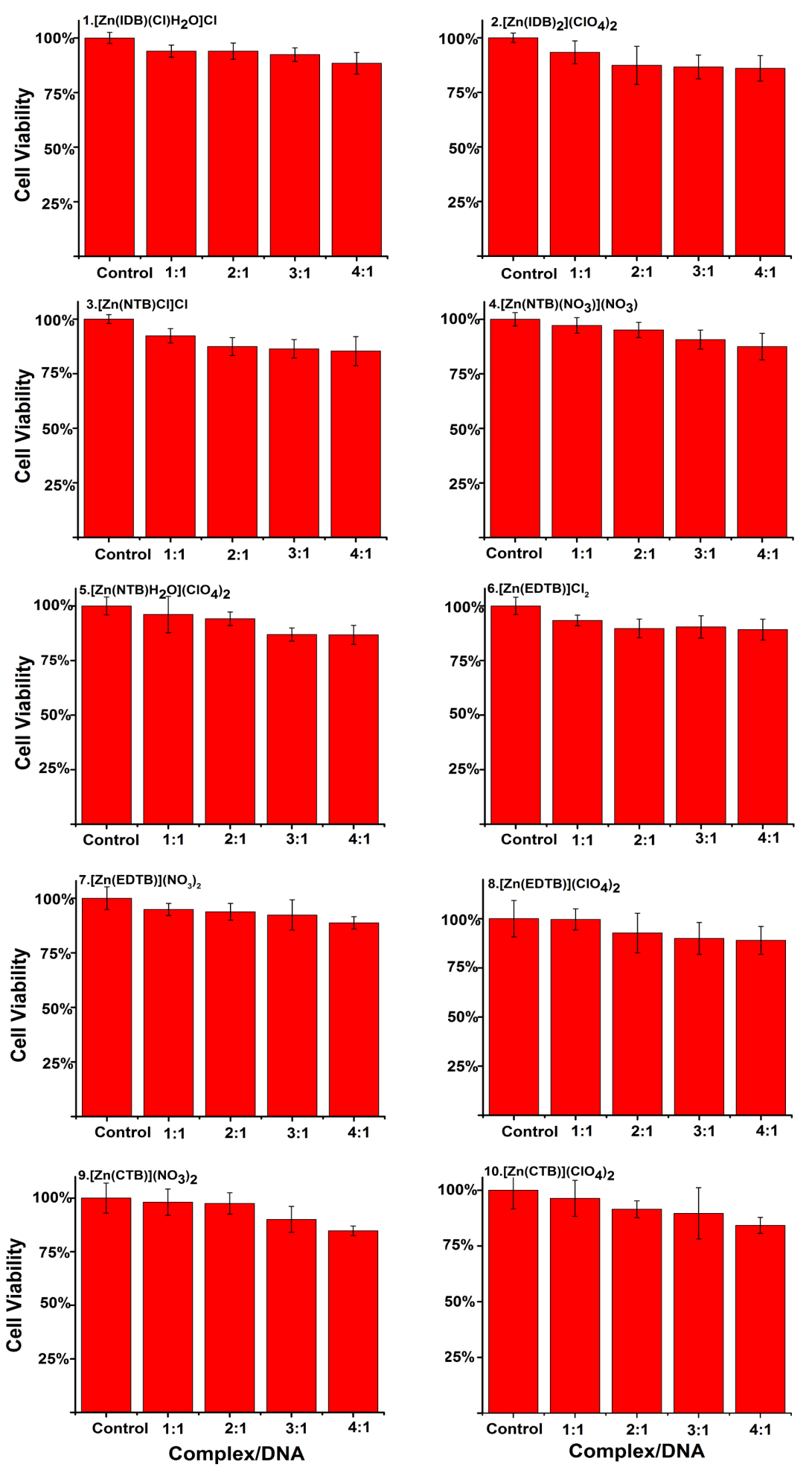

Supplement: S3 Fig — The condensates were prepared, respectively, at the complex/DNA ratios of 1:1, 2:1, 3:1 and 4:1 under the conditions tested. The viability of the COS 7 cells exposed to the condensates was evaluated by MTT assays. (DOCX) [file pone.0158766.s003.docx]

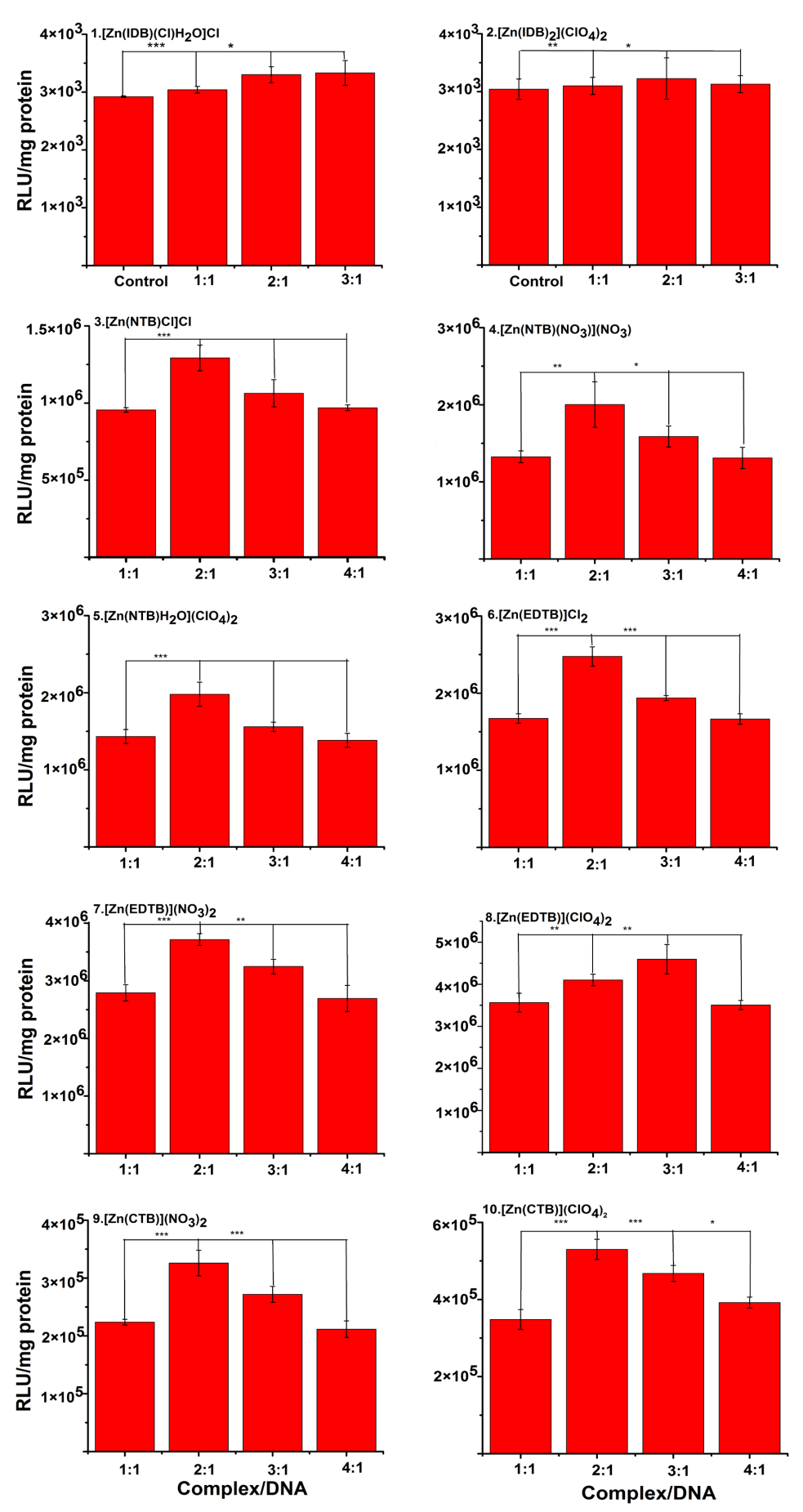

Supplement: S4 Fig — The cell transfection efficacy was expressed by luciferase activity measured in RLU/mg protein. The condensates were prepared at 1:1, 2:1, 3:1 and 4:1 of Zn2+-bzim complex/DNA under the conditions tested. The control was the untreated DNA. n≥ 3, *P = 0.05, **P = 0.01, ***P = 0.001. (DOCX) [file pone.0158766.s004.docx]

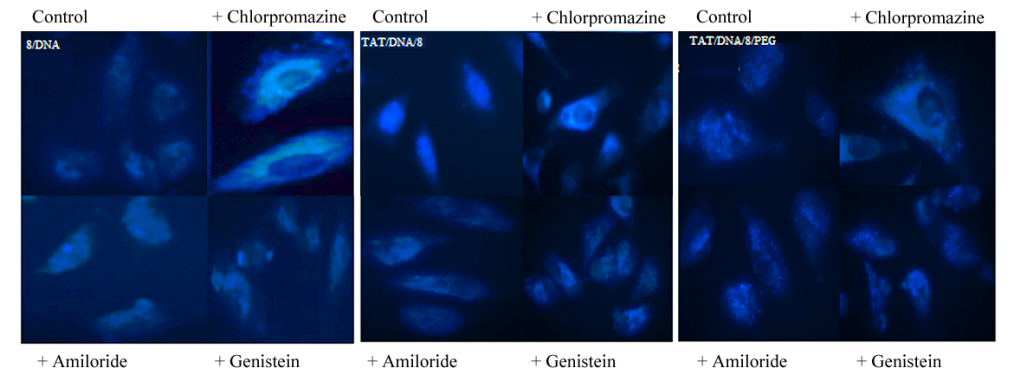

Supplement: S5 Fig — Here, ctDNA was first stained with the fluorescent dye DAPI. Then, the condensates were prepared using the DAPI-stained ctDNA as in Cell Transfection Experiments. These condensates emitted blue fluorescence under fluorescent microscope. (DOCX) [file pone.0158766.s005.docx]
