# Supplementary material for: A Simple Zn2+ Complex-Based Composite System for Efficient Gene Delivery
Source: PLoS One. 2016 Jul 19;11(7):e0158766. doi: 10.1371/journal.pone.0158766 (PMC4951035; doi:10.1371/journal.pone.0158766)
Supplement: S1 Text — (DOCX) [file pone.0158766.s006.docx]

The ligands IDB (bis(benzimidazol-2-ylmethyl)amine), NTB (tris(benzimidazol-2-ylmethyl)amine), EDTB (N,N,N′,N′-tetrakis(benzimidazol-2-ylmethyl)-1,2-ethanediamine), and CTB N,N,N′,N′-tetrakis(2′-benzimidazolylmethyl)-ortho-diamine-trans-cyclohexanewere synthesized and characterized according to the previously reported methods.

[Zn(IDB)Cl(H_2_O)]Cl🞄CH_3_OH,**1**. IDB (0.278 g, 1mmol) was mixedwith ZnCl_2_ (0.150 g, 1.1mmol) in 50 mL methanol.The resulted solution was stirred for 4 h at 70−80^o^C, and filtered following cooling. The filtrate wasallowed to undergo vaporization at room temperature withoutdisturbing.After one week, crystals of **1**were obtained, which lost solvent molecules quickly whenexposed to air.Suitable crystal was selected and sealed in acapillary for X-ray diffraction. Elemental analysis (%),calcd: C 44.03, H 4.36, N 15.10; found: C 38.61, H 4.91,N 14.85.

[Zn(IDB)_2_](ClO_4_)_2_🞄4H_2_O, **2**. IDB (0.584 g, 2.1mmol) was mixedwith Zn(ClO_4_)_2_🞄6H_2_O (0.372 g, 1mmol) in 50 mL methanol.The resulted solution was first stirred for 4 h at 70−80^o^C, and filtered following cooling. The filtrate wasallowed to undergo vaporization at room temperature withoutdisturbing.After one week, crystals of **2**were obtained, which lost solvent molecules quickly whenexposed to air.Suitable crystal was selected and sealed in acapillary for X-ray diffraction. Elemental analysis (%),calcd: C 47.05, H 3.45, N 17.15; found: C 47.24, H 3.65,N 17.92.

[Zn(NTB)Cl]Cl🞄CH_3_OH, **3**. NTB (0.410 g, 1mmol) was mixedwith ZnCl_2_(0.150 g, 1.1mmol) in 50 mL methanol.The resulted solution was first stirred for 4 h at 70−80^o^C, and filtered following cooling. The filtrate wasallowed to undergo vaporization at room temperature withoutdisturbing.After one week, crystals of **3**were obtained, which lost solvent molecules quickly whenexposed to air.Suitable crystal was selected and sealed in acapillary for X-ray diffraction. Elemental analysis (%), calcd: C 49.77, H 3.65, N 16.93; found: C 49.90, H 3.85,N 17.34.

[Zn(NTB)(NO_3_)]NO_3_🞄3CH_3_OH, **4**. NTB (0.410 g, 1mmol) was mixedwith Zn(NO_3_)_2_🞄6H_2_O (0.327 g, 1.1mmol) in 50 mL methanol.The resulted solution was first stirred for 4 h at 70−80^o^C, and filtered following cooling. The filtrate wasallowed to undergo vaporization at room temperature withoutdisturbing.After one week, crystals of **4** were obtained, which lost solvent molecules quickly whenexposed to air.Suitable crystal was selected and sealed in acapillary for X-ray diffraction. Elemental analysis (%), calcd: C 46.79, H 4.80, N18.19; found: C 46.04, H 4.91,N 20.58.

[Zn(NTB)H_2_O](ClO_4_)🞄4CH_3_OH🞄3H_2_O, **5**. NTB (0.410 g, 1mmol) was mixedwith Zn(ClO_4_)_2_🞄6H_2_O (0.409 g, 1.1mmol) in 50 mL methanol.The resulted solution was first stirred for 4 h at 70−80^o^C, and filtered following cooling. The filtrate wasallowed to undergo vaporization at room temperature withoutdisturbing.After one week, crystals of **5**were obtained, which lost solvent molecules quickly whenexposed to air.Suitable crystal was selected and sealed in acapillary for X-ray diffraction. Elemental analysis (%), calcd: C 41.79, H 3.36, N14.21; found: C 42.73, H 4.01,N 14.90.

[Zn(EDTB)]Cl_2_🞄3CH_3_OH, **6**. EDTB (0.588 g, 1mmol) was mixedwith ZnCl_2_(0.150 g, 1.1mmol) in 50 mL methanol.The resulted solution was first stirred for 4 h at 70−80^o^C, and filtered following cooling. The filtrate wasallowed to undergo vaporization at room temperature withoutdisturbing.After one week, crystals of **6**were obtained, which lost solvent molecules quickly whenexposed to air.Suitable crystal was selected and sealed in acapillary for X-ray diffraction. Elemental analysis (%), calcd: C 54.58, H 5.57, N 17.20; found: C 52.09, H 4.91,N 18.63.

[Zn(EDTB)](NO_3_)_2_🞄CH_3_OH, **7**. EDTB (0.588 g, 1mmol) was mixedwith Zn(NO_3_)_2_🞄6H_2_O (0.327 g, 1.1mmol) in 50 mL methanol.The resulted solution was first stirred for 4 h at 70−80^o^C, and filtered following cooling. The filtrate wasallowed to undergo vaporization at room temperature withoutdisturbing.After one week, crystals of **7**were obtained, which lost solvent molecules quickly whenexposed to air.Suitable crystal was selected and sealed in acapillary for X-ray diffraction. Elemental analysis (%), calcd: C 53.03, H 4.91, N21.83; found: C 53.03, H 4.87,N 21.86.

[Zn(EDTB)](ClO_4_)_2_🞄CH_3_OH🞄CH_3_CH_2_OCH_2_CH_3_, **8**. EDTB (0.588 g, 1mmol) was mixedwith Zn(ClO_4_)_2_🞄6H_2_O (0.409 g, 1.1mmol) in 50 mL methanol.The resulted solution was first stirred for 4 h at 70−80^o^C, and filtered following cooling. The filtrate wasallowed to undergo vaporization at room temperature withoutdisturbing.After one week, crystals of **8**were obtained, which lost solvent molecules quickly whenexposed to air.Suitable crystal was selected and sealed in acapillary for X-ray diffraction. Elemental analysis (%), calcd: C 48.33, H 3.82, N16.58; found: C 49.16, H 3.91,N 17.35.

[Zn(CTB)](NO_3_)_2_🞄CH_3_OH, **9**. NTB (0.634 g, 1mmol) was mixedwith Zn(NO_3_)_2_🞄6H_2_O (0.327 g, 1.1mmol) in 50 mL methanol.The resulted solution was first stirred for 4 h at 70−80^o^C, and filtered following cooling. The filtrate wasallowed to undergo vaporization at room temperature withoutdisturbing.After one week, crystals of **9**were obtained, which lost solvent molecules quickly whenexposed to air.Suitable crystal was selected and sealed in acapillary for X-ray diffraction. Elemental analysis (%), calcd: C 54.71, H 4.94, N19.63; found: C 55.65, H 4.91,N 20.98.

[Zn(CTB)](ClO_4_)_2_🞄CH_3_OH, **10**. NTB (0.634 g, 1mmol) was mixedwith Zn(ClO_4_)_2_🞄6H_2_O (0.409 g, 1.1mmol) in 50 mL methanol.The resulted solution was first stirred for 4 h at 70−80^o^C, and filtered following cooling. The filtrate wasallowed to undergo vaporization at room temperature withoutdisturbing.After one week, crystals of **10**were obtained, which lost solvent molecules quickly whenexposed to air.Suitable crystal was selected and sealed in acapillary for X-ray diffraction. Elemental analysis (%), calcd: C 50.31, H 4.55, N15.04; found: C 51.35, H 4.61,N 16.43.
